# Mechanism of Action of Two Flavone Isomers Targeting Cancer Cells with Varying Cell Differentiation Status

**DOI:** 10.1371/journal.pone.0142928

**Published:** 2015-11-25

**Authors:** Timothy M. LeJeune, Hei Yin Tsui, Laura B. Parsons, Gerald E. Miller, Crystal Whitted, Kayla E. Lynch, Robert E. Ramsauer, Jasmine U. Patel, Jarrett E. Wyatt, Doris S. Street, Carolyn B. Adams, Brian McPherson, Hei Man Tsui, Julie A. Evans, Christopher Livesay, Ruben D. Torrenegra, Victoria E. Palau

**Affiliations:** 1 Bill Gatton College of Pharmacy, East Tennessee State University, Johnson City, TN, 37614, United States of America; 2 Universidad de Ciencias Aplicadas y Ambientales, Bogota, Colombia; 3 Division of Hematology-Oncology, Department of Internal Medicine, James Quillen College of Medicine, East Tennessee State University, Johnson City, TN, 37614, United States of America; 4 Department of Pharmaceutical Sciences, Bill Gatton College of Pharmacy, East Tennessee State University, Johnson City, TN, 37614, United States of America; Taipei Medicine University, TAIWAN

## Abstract

Apoptosis can be triggered in two different ways, through the intrinsic or the extrinsic pathway. The intrinsic pathway is mediated by the mitochondria via the release of cytochrome C while the extrinsic pathway is prompted by death receptor signals and bypasses the mitochondria. These two pathways are closely related to cell proliferation and survival signaling cascades, which thereby constitute possible targets for cancer therapy. In previous studies we introduced two plant derived isomeric flavonoids, flavone A and flavone B which induce apoptosis in highly tumorigenic cancer cells of the breast, colon, pancreas, and the prostate. Flavone A displayed potent cytotoxic activity against more differentiated carcinomas of the colon (CaCo-2) and the pancreas (Panc28), whereas flavone B cytotoxic action is observed on poorly differentiated carcinomas of the colon (HCT 116) and pancreas (MIA PaCa). Apoptosis is induced by flavone A in better differentiated colon cancer CaCo-2 and pancreatic cancer Panc 28 cells via the intrinsic pathway by the inhibition of the activated forms of extracellular signal-regulated kinase (ERK) and pS6, and subsequent loss of phosphorylation of Bcl-2 associated death promoter (BAD) protein, while apoptosis is triggered by flavone B in poorly differentiated colon cancer HCT 116 and MIA PaCa pancreatic cancer cells through the extrinsic pathway with the concomitant upregulation of the phosphorylated forms of ERK and c-JUN at serine 73. These changes in protein levels ultimately lead to activation of apoptosis, without the involvement of AKT.

## Introduction

The prevalence of cancer has steadily increased throughout the past decades [1]. While current therapies are effective at various levels, many of these treatments are also nonspecific with respect to their mechanism of action. This in turn increases the undesirable outcomes experienced by treated patients; harsh side effects and low rates of effectiveness are some of the many reasons why more specific treatments in oncology have been sought. Among these efforts, natural products[2] have been studied extensively in hopes of identifying new molecular entities with antineoplastic properties. Of these natural products, flavonoids, one class of polyphenolic compounds found in plants, have been shown to exert antineoplastic [3–9] properties, as well as antioxidant [10, 11], anti-inflammatory [12], antimicrobial [13] and antiviral [14, 15] activities.

For the past five years our research has focused on plants from the Andean mountains largely known as *vira viras*. These plants have been utilized by the native people of this region for medicinal purposes such as in the treatment of cancer, as reported in various ethnobotanical studies [16, 17]. They belong to the family *Asteraceae*, genera *Gnaphalium*, *Achyrocline*, and *Gamochaeta*. Our focus with respect to vira vira plants has been placed on *Gnaphalium elegans* and *Achyrocline bogotensis* from which two active compounds were isolated, 5,7-dihydroxy 3,6,8-trimethoxy-2-phenyl-4H-chromen-4-one (5,7-dihydroxy-3,6,8-trimethoxyflavone or flavone A) [18], and 3,5-dihydroxy-6,7,8-trimethoxy-2- phenyl-4H-chromen-4-one (3,5-dihydroxy-6,7,8-trimethoxyflavone or flavone B) respectively [19]. It has been proposed in our previous work that these two flavone isomers may be relevant to the antineoplastic activities of these plants [20]. Indeed, flavones A and B demonstrated cytotoxic activity against cell lines derived from colon, pancreas, breast, and prostate cancers that have been categorized as being highly tumorigenic, with the most promising results on cancers of the colon and pancreas. High levels of expression of aldehyde dehydrogenase (ALDH) is regarded as a very specific marker used in the detection of cancer-initiating cells as subpopulations in tumors, and has been demonstrated specifically in the cell lines studied [21–23]. Among these highly tumorigenic cell lines, the two flavone isomers show preferential antineoplastic activity on cells with dissimilar differentiation status. Flavone A induced apoptosis in the better differentiated cell lines, but not on the poorly differentiated cell lines, while flavone B was shown to be active against poorly differentiated, but not against the better differentiated cells. Moreover, these two flavone isomers do not induce apoptosis in normal cells and display significantly less apoptotic activity in less tumorigenic cell lines [20].

It is known that mitochondrial management of apoptosis can be controlled through the activity of survival factors, such as growth factors or cytokines, via extracellular receptors activating cascades of protein events that eventually lead to Caspase-3 cleavage. These pathways were probed by investigating the effects of the two flavone isomers on extracellular signal-regulated kinases (ERK), protein kinase B (AKT), S6 ribosomal protein (S6), and Bcl-2 associated death promoter (BAD). The preferential induction of apoptosis on highly tumorigenic cells with dissimilar differentiation status by flavone A and flavone B, two structurally similar compounds, suggest the activation of different cellular pathways by each compound. This study shows that flavone A exerts its cytotoxic effect on better differentiated cancer cells via an intrinsic apoptotic pathway whereas flavone B bypasses the mitochondrial pathway to induce apoptosis via an extrinsic pathway in poorly differentiated cancer cells.

## Materials and Methods

### Extraction, Purification, and Identification of Flavones

The flavones were obtained as described before [20]. Briefly, flavone A was purified from 1.5 kg of *G*. *elegans* dried flowers extracted with CHCl_3_. The extract was concentrated by dry vacuum, dissolved in methanol, and filtered to eliminate fats and hydrocarbons. It was then concentrated and dissolved in C_6_H_6_ followed by silica gel chromatography using C_6_H_6_:Me_2_CO (19:1) as eluent. From this, 50 mg of the flavonoid was purified from fractions 12 through 18 by crystallizations in hexane. The compound was identified by its physical and spectroscopic properties as 5,7 dihydroxy-3,6,8 trimethoxyflavone, mp 170 171C, 1H NMR (300MHz) 3.86 (3H,s), 3.97 (3H,s), 4.20 (3H,s), 7.50 7.6 (3H,m), 8.08 8.16 (2H,m). Flavone B was purified from 200 g of fresh leaves of *A*. *bogotensis*. The leaves were submerged in CHCl_3_ for 20 minutes and then filtered, concentrated, and dissolved in hot methanol. In order to eliminate fats and hydrocarbons, the cold extract was filtered and concentrated again. The resulting solid was then dissolved in hot hexane and successive recrystallizations in hexane produced 100 mg of purified flavonoid. The compound was identified by its physical and spectroscopic properties as 3,5-dihydroxy-6,7,8-trimethoxyflavone, mp 149 150C, 1H NMR (300MHz) 3.86 (3H,s), 3.97 (3H,s), 3.99 (3H,s), 4.12 (3H,s), 7.30 7.45 (3H,m), 8.70 8.82 (3H,m), 11.46 (1H,s).

#### Cell Lines and Culture Conditions

Colon (CaCo-2, HCT116) and pancreatic (MIA PaCa-2) cell lines were obtained from the American Type Culture Collection (ATCC, Manassas, VA) and were grown according to ATCC instructions. The Panc28 cell line was a gift from Dr. Paul Chiao (University of Texas M.D. Anderson Cancer Center, Houston, TX), and was grown in the same manner as pancreatic cell line MIA Paca-2. The cells were grown in media supplemented with 10% serum (Gibco, Grand Island, NY) and penicillin/streptomycin (Hyclone, Logan, UT). All cells were seeded and allowed to reach 75% confluency before treatment with flavone A, B, or vehicle (dimethyl sulfoxide) at a final maximum concentration of 0.27% in the treated wells (Sigma Aldrich, St Louis, MO).

### Apoptosis Detection by Fluorescence Microscopy

Apoptosis was detected using the Annexin V-FITC kit from Abcam (Cambridge, MA) according to manufacturer instructions. Briefly, cells were seeded at a density of 4-5x10^4^/well on 12-mm round coverslips (Fisher Scientific, Pittsburgh, PA), allowed to reach 75% confluence, and treated with either vehicle, Flavone A, or Flavone B at a concentration of 40 μM. Six hours after dosing, the cells were incubated in binding buffer and FITC-conjugated Annexin V for five minutes in the dark. The cells were then fixed with 2% p-formaldehyde and images were obtained using an EVOS fluorescence microscope (AMG, Bothell, WA).

### Cell Cycle and Apoptosis Analysis by Flow Cytometry

Cells grown on six well plates, were treated with either dissolution vehicle, flavone A, or flavone B. After nine hours of incubation, the cells were lifted from the plate with trypsin, and assayed for quantitation of apoptosis and cell cycle distributions using an Annexin V-FITC kit from Abcam (Cambridge, MA) according to the manufacturer instructions. Briefly, the cells were suspended in binding buffer and Annexin V-FITC and propidium iodide were added, followed by a 5 minute incubation in the dark. Cell count was obtained using BD Accuri C6 flow cytometer (BD Biosciences, San Jose, CA) and analyzed with CFLOW Plus (BD Biosciences).

### Antibodies and Reagents

The mechanism of action was assessed using antibodies against ERK2 from Santa Cruz (Dallas TX), AKT from Millipore (Billerica, MA), c-JUN and BAD from Cell Signaling (Beverly, MA), phosphorylated c-JUN (T91/93) from Abcam (Cambridge, MA), phosphorylated c-JUN (S63/73), the phosphorylated forms of ERK, S6 ribosomal protein (91B2), and BAD, from Cell Signaling, phosphorylated AKT S473 from Millipore, BH3-interacting domain death agonist (Bid) from R&D Systems (Minneapolis, MN), inactive and active forms of caspase 3 from Santa Cruz and R&D Systems, caspase 8 and caspase 10 from MBL (Woburn, MA), and caspase 9 from Cell Signaling, and α-tubulin and β-actin from Sigma (St. Louis, MO). All secondary antibodies were affinity purified with no cross-reactivity with other species. Peroxidase-conjugated secondary antibodies were obtained from Pierce (Rockford, IL), Promega (Madison, WI), and Jackson ImmunoResarch (West Grove, PA). Alexa Fluor 488 and 594-conjugated secondary antibodies (Molecular Probes, Eugene, OR) were used as specified by the manufacturer. Tamoxifen used to obtain a positive control signal for the phosphorylation analysis of c-JUN was from Sigma.

### PAGE and Immunoblot

Treated cells were lysed with lysis buffer which consisted of 20 mM imidazole, 100 mM KCl, 1mM MgCl_2_, 10mM EGTA, 0.2% Triton X-100, phosphatase, and protease inhibitors (Sigma Aldrich). Protein concentration of cell lysates was measured spectrophotometrically (Cary 50; Varian, Palo Alto, CA) using a protein assay from Cytoskeleton (Denver, CO, USA). The samples were run in SDS-PAGE and then blotted onto nitrocellulose or PVDF sheets. The signal of the primary monoclonal or polyclonal antibodies was detected using secondary affinity-purified goat anti-mouse or anti-rabbit immunoglobulins coupled to peroxidase and a chemiluminescent system (Pierce; Grand Island, NY) and exposed on x-ray film (Kodak; Rochester, NY). The intensity of the bands was estimated by digitizing the image (J- Image) from x-ray film. After subtracting the background, all band intensities were compared against control.

### Immunofluorescence

Immunofluorescence was carried out as described before [24]. Briefly, cells were fixed with 3% p-formaldehyde for 20 min at room temperature. After rinsing, the cells were permeabilized with 0.2% Triton X-100 for 5 min or 0.1% saponin throughout the procedure. Permeabilization was followed by quenching of the aldehyde groups in 50 mM NH_4_Cl. Cells were then incubated with primary antibody diluted in 1% BSA, for 1 hour at room temperature. After washes and incubation with secondary antibody, the cells were mounted in 10% polyvinyl alcohol, 30% glycerol, 1% n-propyl gallate, and Slow Fade (Molecular Probes, Invitrogen) at a dilution of 5:1. Fluorescence images were obtained using an EVOS fluorescence microscope (AMG, Bothell, WA).

## Results

### Flavone A and Flavone B induce apoptosis and a cell cycle shift

**Fig 1:**
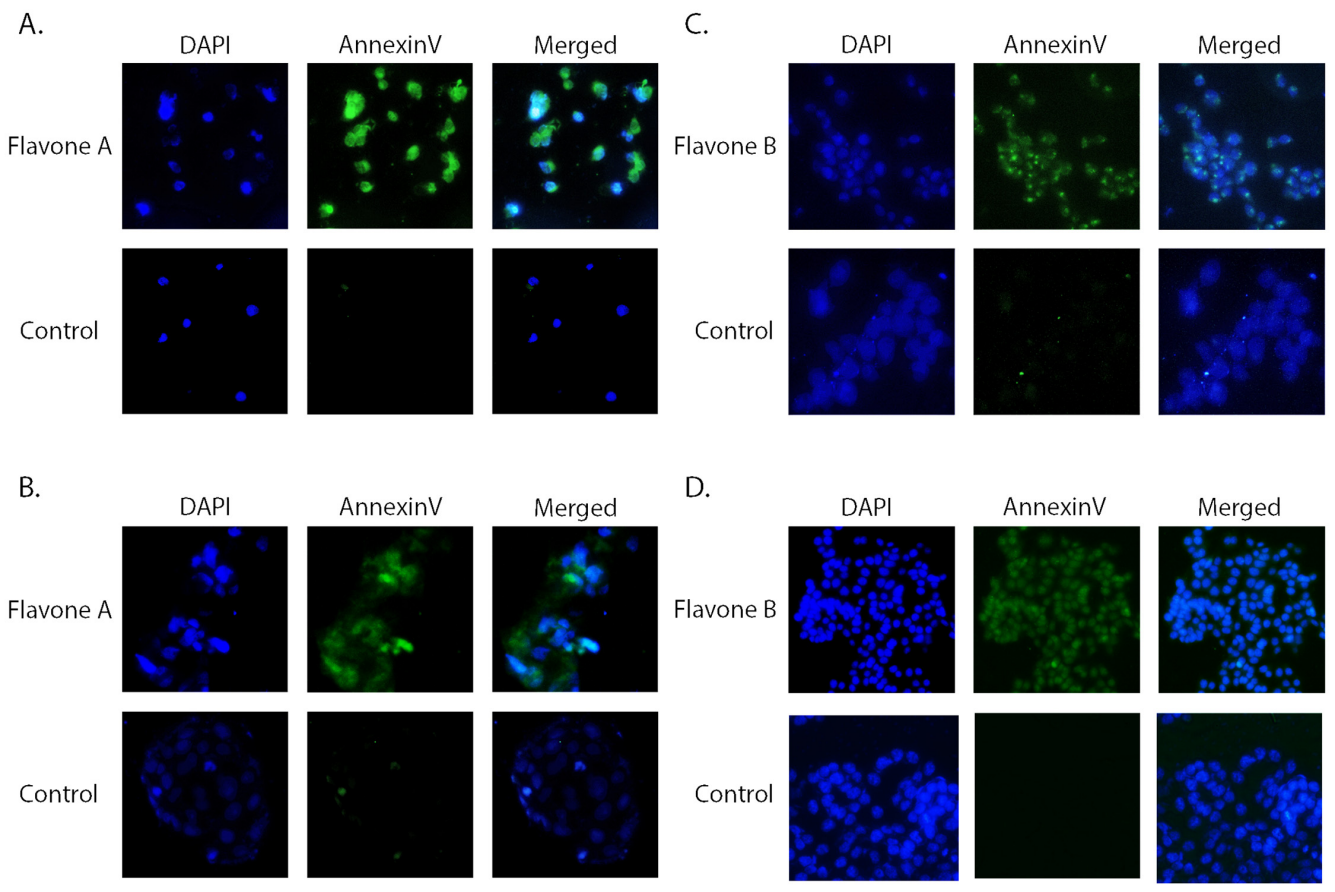
Annexin V assay. Apoptotic effect of flavone A at a concentration of 40 μM, on the more differentiated pancreatic Panc28 and colon CaCo 2 cancer cells (Fig 1A and 1B), as determined by Annexin V assay (green channel) six hours after treatment. Dapi (blue channel) is used to locate the nuclei of the cells. Cells treated with vehicle only (DMSO at a final concentration of 0.27%) served as a control. Activation of apoptosis on the poorly differentiated pancreatic MIA PaCa and colon HCT116 cancer cells (Figs 1C and 1D) by flavone B at a concentration of 40 μM, as determined by Annexin V assay (green channel) six hours after treatment. Control conditions are the same as described above and Dapi was used to locate nuclei.

**Fig 2:**
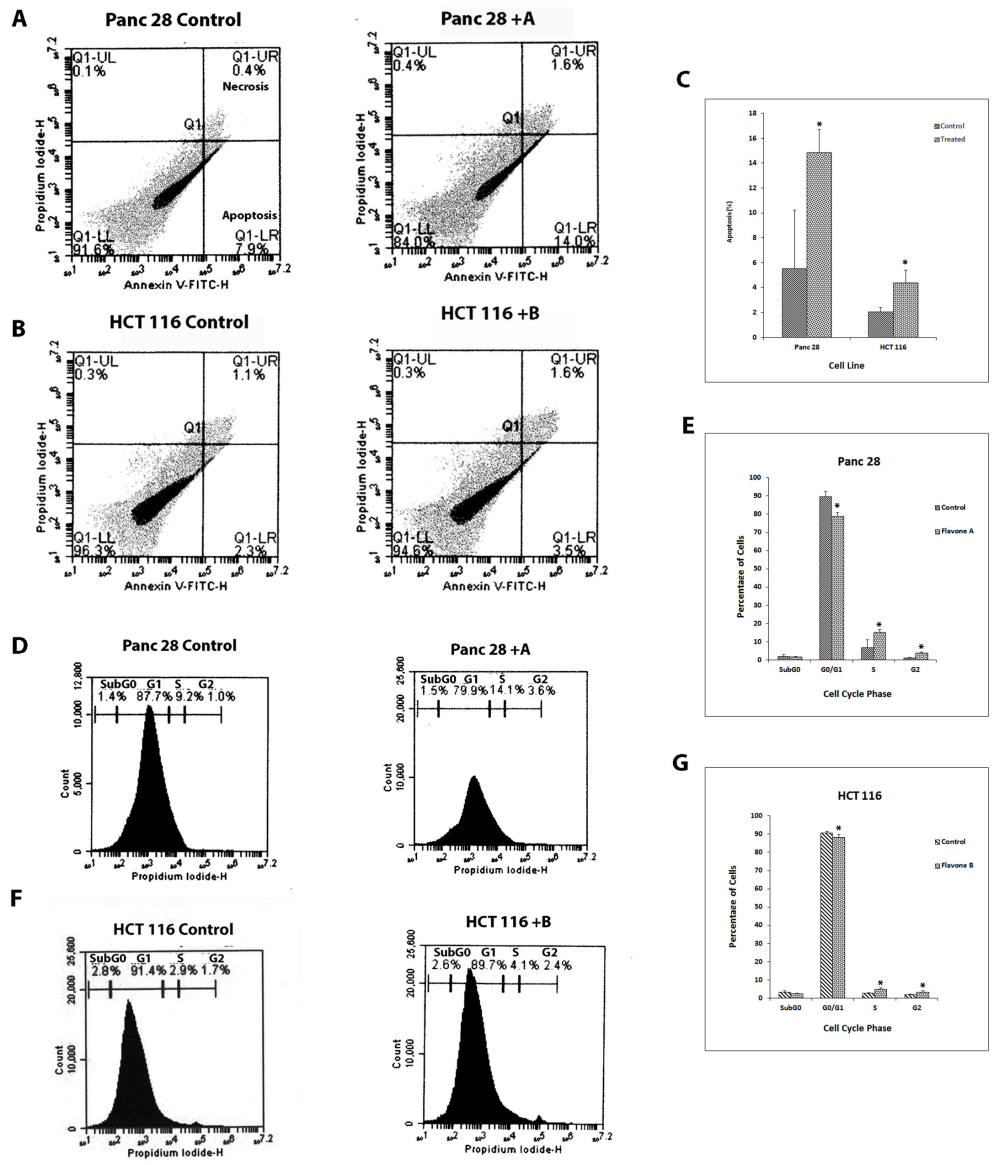
Annexin V-FITC and propidium iodide flow cytometry. A. Apoptosis was detected using Annexin V-FITC and propidium iodide in Panc 28 cells treated with 40 μM flavone A, 9 hours after treatment. B. Detection of apoptosis in HCT 116 cells treated with 40 μM flavone B, 9 hours after treatment. C. Bar graph representation of apoptosis in Panc 28 and HCT 116 cells treated with flavone A and B respectively. D-E. Cell cycle determination using propidium iodide in Panc 28 cells treated 40 μM flavone A. F-G. Cell cycle determination in HCT 116 cells treated with 40 μM flavone B.

Our previous data suggest that flavones A and B cause DNA fragmentation on better and poorly differentiated cells respectively [20]. Annexin V assays were conducted to confirm the induction of apoptosis on tumorigenic cells status after dosing with the flavones. Apoptosis was detected 6 hours after pancreatic cancer Panc-28 (Fig 1A) and colon cancer Caco-2 (Fig 1B) cells were dosed with 40 μM of flavone A. In the same manner, apoptosis was detected 6 hours after pancreatic cancer MIA Paca-2 (Fig 1C) and colon cancer HCT116 cells (Fig 1D) were dosed with 40 μM of flavone B. To quantify the apoptotic changes induced by the flavones, cells dosed with the vehicle or flavone, were analyzed 6, 9 and 12 hours after treatment via flow cytometry using Annexin V/ propidium iodide. At 9 hours, Panc 28 cells treated with flavone A had 2.9 fold increase in apoptosis (Fig 2A and 2C). Similarly, at 9 hours, HCT 116 cells treated with flavone B had a 2.3 fold increase in apoptosis (Fig 2B and 2C).

To test whether treatment with flavone A or flavone B had an effect on cell cycle distributions, flow cytometry assays using propidium iodide were performed. Better differentiated pancreatic cells Panc 28 cells were treated with flavone A (Fig 2D and 2E). After 9 hours, a shift from the G0/G1 to the S and G2 phases is clearly seen. Poorly differentiated colon cancer cells HCT 116 were treated with flavone B. A similar shift from G0/G1 phase to S and G2 phases is apparent 9 hours after treatment (Fig 2F and 2G).

### Flavone A has an inhibitory effect on phosphorylated ERK and S6, but has no effect on AKT or c-JUN in better differentiated Panc28 and CaCo-2 cancer cells

**Fig 3:**
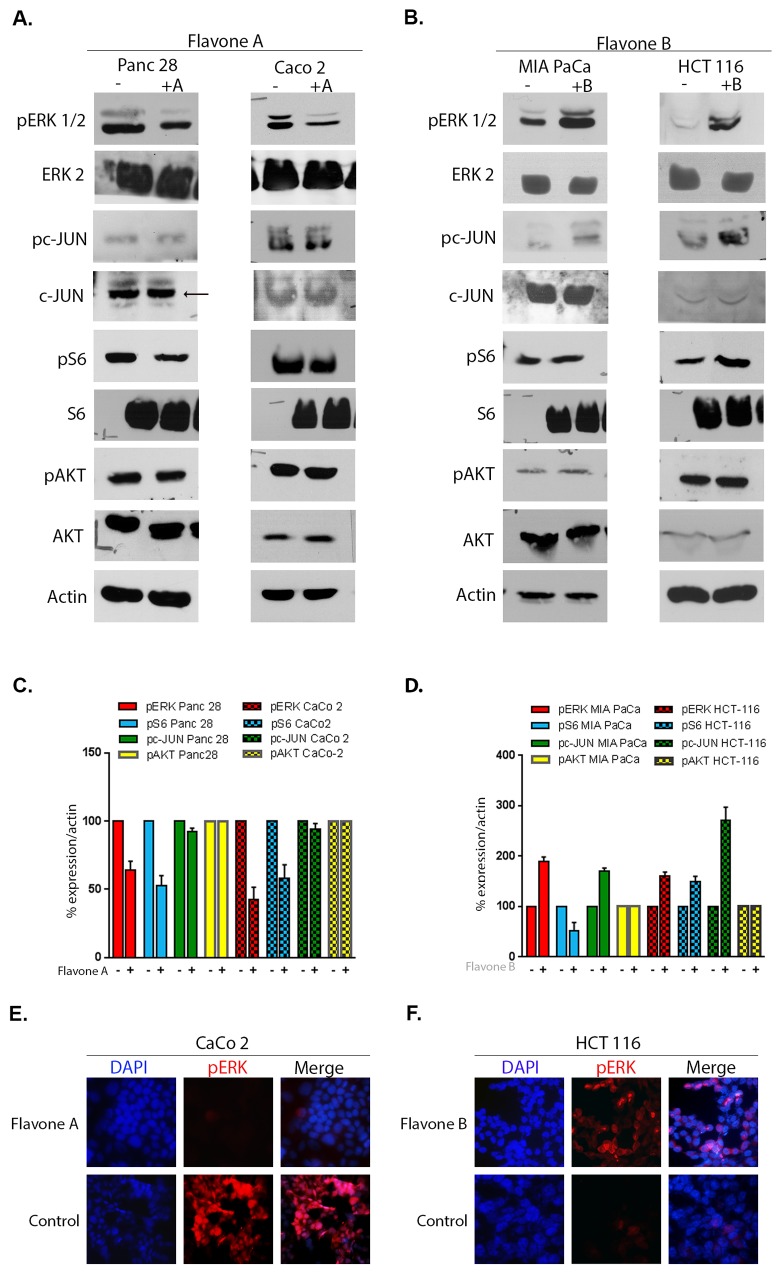
Comparison of the effect of flavone A and flavone B on proliferative, and survival pathways. A and B: Detection of the activated and unphosphorylated forms of ERK, c-JUN, S6, AKT by immunoblot of total SDS extracts. Better differentiated Panc28 and CaCo 2 cells were treated with 40μM of flavone A (+A), and poorly differentiated MIA PaCa and HCT116 cells with flavone B (+B), or DMSO (-) the dissolution vehicle. After lysis and SDS-PAGE, membranes were probed with the indicated antibody. The membranes were reprobed for actin as a loading control, and a representative image is provided. The results shown are representative of three independent experiments. C and D: For quantification (graphs) the band densities from the treated/untreated conditions identified by (+) or (-), were normalized and calculated as percentages of the value for the untreated cells (100%), and shown averages ± standard deviations from three independent experiments (*p<0.05). E and F: Detection of phosphorylated ERK after treatment of CaCo 2 cells with flavone A and HCT116 cells with flavone B by immunofluorescence.

In order to determine the mechanism responsible for the cytotoxic effect on better differentiated cancer cells observed after treatment with flavone A, proliferative, survival, and apoptotic signaling pathways were examined. Colon cancer CaCo-2 and pancreatic cancer Panc28 cells were dosed with 40 μM of flavone A and levels of activated AKT, ERK, S6, and c-JUN were analyzed. As shown in Fig 3A and 3C, dosing Panc 28 cells with flavone A induced a mean reduction of the phosphorylated form of ERK to 64.27% (51.84%–74.83%; p = 0.0029) and of pS6 to 52.58% (37.90%–62.50%; p = 0.012) as compared to the control. In CaCo-2 cells, the mean reduction of phosphorylated ERK by flavone A was to 42.58% (25.38%–55.64%; p = 0.0031), and 57.99% (43.84%–77.36%; p = 0.0138) for pS6, as compared to the control. No change was observed in the expression levels of the unphosphosphorylated proteins analyzed (Fig 3A). These immunoblot results were confirmed by immunofluorescence. Phosphorylated ERK results in CaCo-2 cells are shown in Fig 3E. Furthermore, the activated forms of AKT at serine 473 and c-JUN at serine 73, were also studied as these proteins regulate both cell survival and stress-induced apoptosis respectively. Neither of these demonstrated significant changes in Panc-28 and CaCo-2 cell lines (Fig 3A and 3C).

### Increased expression of the phosphor-c-JUN (S73) and ERK were observed after treatment of poorly differentiated MIA PaCa and HCT116 cancer cells with Flavone B

A mean increase to 190.19% of activated ERK in pancreatic cancer MIA PaCa cells (175.32%–204.08%; p = 0.0036; n = 3), and 160.23% in colon cancer HCT116 cells (144.99%–174.22%; p = 0.012; n = 3) were observed after treatment with flavone B (Fig 3B and 3D) as compared to controls. No change was observed in the expression levels of the unphosphosphorylated proteins analyzed (Fig 3B). These immunoblot results were confirmed by fluorescence microscopy and the phosphorylated ERK results are shown for HCT-116 cells (Fig 3F). Also observed were increased levels of the phosphorylated form of c-JUN (S73) in MIA PaCa at 170.83% from the control levels (162.27%–181.90%; p = 0.0008; n = 3), and in HCT116 at 271.34% (222.95%–312.93%; p = 0.0028; n = 3) as shown in Fig 3B and 3D.

### Flavone B has dissimilar effects on pS6 in MIA PaCa and HCT116 cells, but has no effect on phosphorylated AKT

After treatment of MIA PaCa cells with flavone B, the mean levels of pS6 are decreased to 51.68% (19.95%–77.77%; p = 0.0237; n = 3) versus control as seen in Fig 3D. Conversely, after treatment of HCT116 cells, an increase of 149.88% (136.54–170.22; p = 0.0116; n = 3) is observed, as compared to the controls. The expression levels of phosphoserine 473 AKT, were unchanged after treatment of MIA PaCa and HCT116 cells with flavone B as shown in Fig 3B and 3D.

### Flavone A modulates BAD phosphorylation at serine 112 but not flavone B

**Fig 4:**
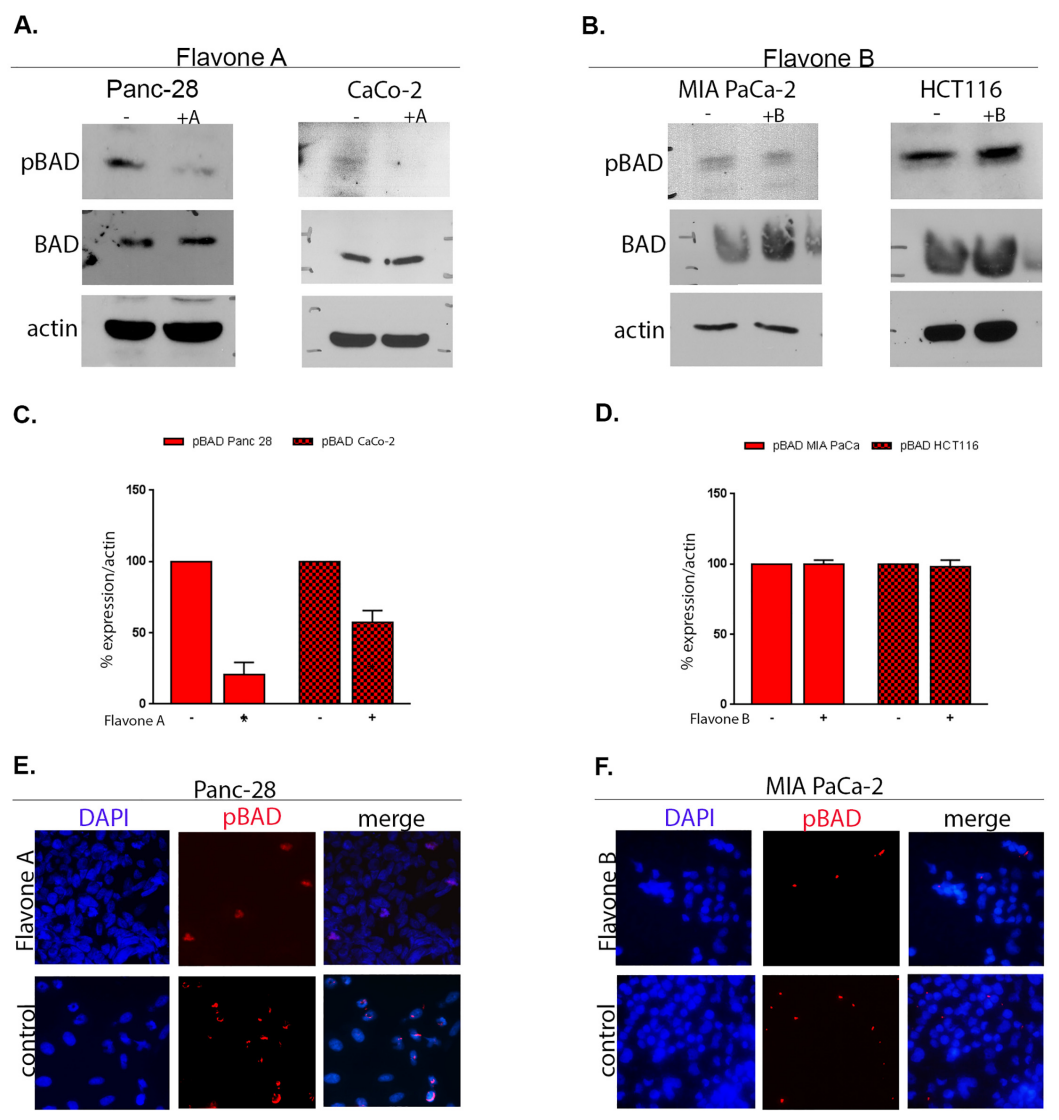
Analysis of downstream effector BAD after treatment with flavone A and flavone B. A and B: Detection of the loss of phosphorylation of BAD by immunoblot of total SDS extracts. Better differentiated Panc 28 and CaCo 2 cells were treated with 40μM of flavone A (+A), and poorly differentiated MIA PaCa and HCT116 cells with flavone B (+B), or DMSO (-) the dissolution vehicle. After lysis and SDS-PAGE, membranes were probed with an antibody specific to BAD phosphorylated at serine 112 or the unphosphorylated protein. The membranes were reprobed for actin as a loading control. The results shown are representative of three independent experiments. C and D: For quantification (graphs) the band densities from the treated/untreated conditions identified by (+) or (-), were normalized and calculated as percentages of the value for the untreated cells (100%), and shown averages ± standard deviations from three independent experiments (*p<0.05). E and F: Detection of phosphorylated BAD at serine 112 (red channel), after treatment of Panc 28 cells with flavone A and MIA PaCa cells with flavone B by immunofluorescence. Dapi (blue channel) was used to locate the nuclei.

To further investigate the differences of cellular effect of flavone A and flavone B, the phosphorylation status of BAD at serine 112 was studied. Cell lines Panc-28 and CaCo-2 dosed with flavone A exhibit a decline in phosphorylated BAD at serine 112 (Fig 4A). This reduction had a mean of 35.91% (34.21%–37.61%; p = 0.006; n = 3) in Panc-28, and 57.03% (42.12%–71.62%; p = 0.0068; n = 3) in CaCo-2 cells (Fig 4C). These observations were confirmed via immunofluorescence for Panc-28 as shown in Fig 4E. Conversely, dosing MIA PaCa-2 and HCT116 tumorigenic cells with 40 μM of flavone B produced no significant change in the total amount of phosphorylated BAD at serine 112 as shown by western blot (Fig 4B and 4D) and immunofluorescence for MIA Paca-2 (Fig 4F). While there is no significant change observed in the expression levels of unphosphorylated BAD in better differentiated cells (Fig 4A), a slight increase is observed in the poorly differentiated cells (Fig 4B).

### Flavone A may induce apoptosis via caspase 9

**Fig 5:**
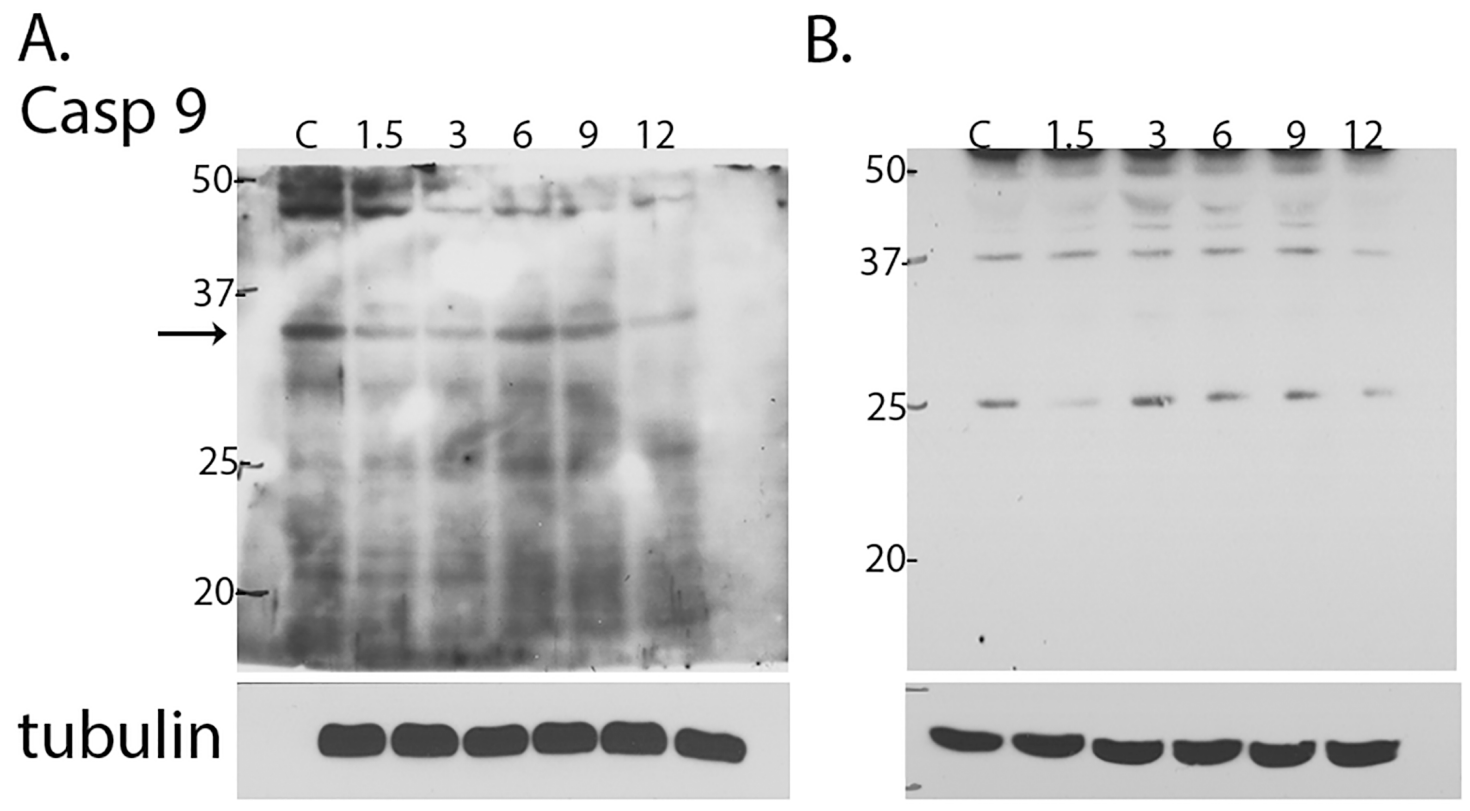
Analysis of caspase 9 after treatment with flavone A. Detection of activated caspase 9 by immunoblot of SDS extracts of A. CaCo 2 and B. Panc 28 cells 1.5, 3, 6, 9 and 12 hours (lanes 2–6) after treatment with flavone A or vehicle (DMSO) for the control (C, lane 1) and SDS-PAGE. The membranes were probed with an antibody capable of detecting both the procaspase (47 kDa) and the large fragments resultant after activation (37 and 35 kDa). The membranes were reprobed for actin or tubulin as a loading control. The results shown are representative of three independent experiments. The membranes were reprobed for actin or tubulin as a loading control. The results shown are representative of three independent experiments.

To determine whether caspase 9 participates in the apoptotic cascade initiated by flavone A, CaCo-2 and Panc28 cells were dosed and analyzed by SDS PAGE followed by immunoblot for the presence of cleaved fragments of 37 and 17 kDa with an antibody capable of detecting the activated caspase. Fig 5A shows a representative immunoblot of CaCo-2, and Fig 5B for Panc28 cells of lysates taken at different time points after dosing with flavone A. Both cell lines display the large fragments, 37 and 35 kDa, detected by the antibody (Fig 5A and 5B). The 37 kDa fragment is apparent in the control (cells dosed with vehicle) suggesting deregulation of the protein in these cell lines. However, a progressive reduction in the level of expression of procaspase 9 (47kDa) is evident starting 3 hours after dosing.

### Flavone B neither phosphorylate c-JUN at threonines 91 and 93 nor activate caspases 8 and 10 in HCT 116 or MIA PaCa cells

**Fig 6:**
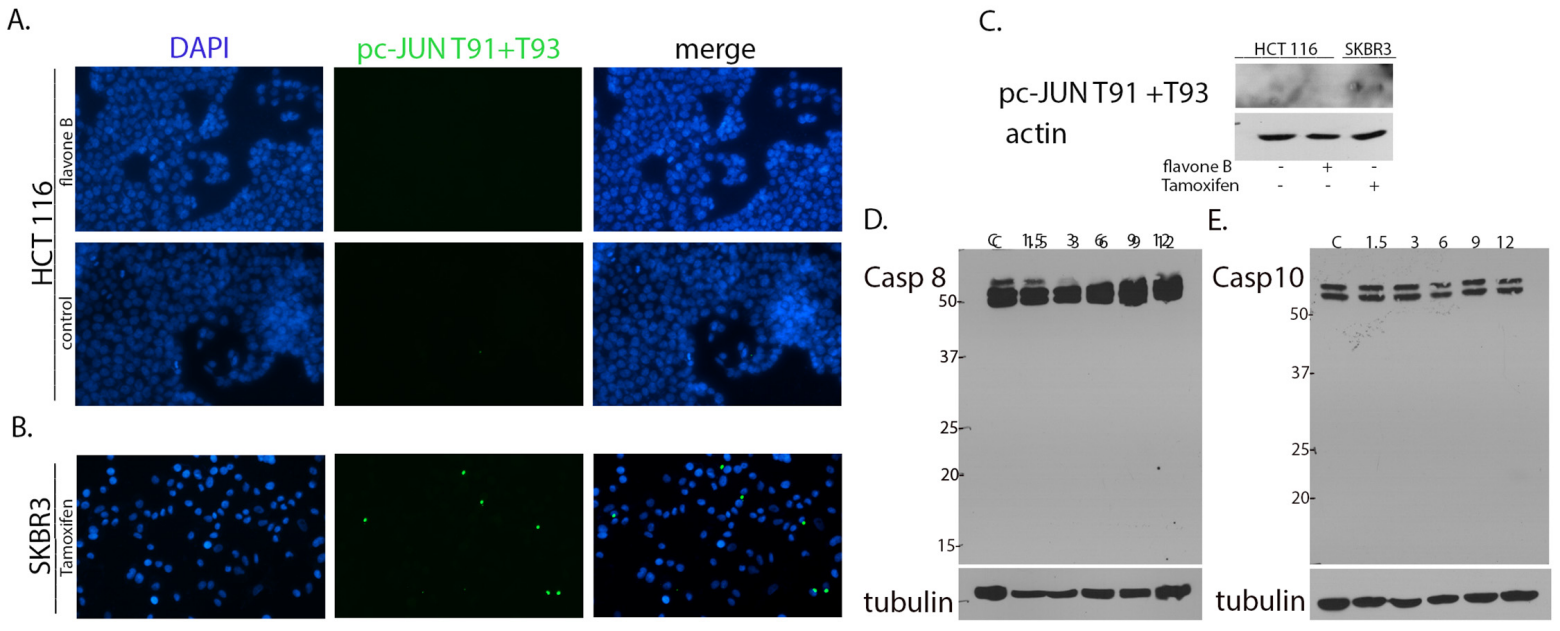
Analysis of c-JUN phosphorylation of threonines 91/93 and caspase 8 after treatment with 40 μM flavone B. A and B. Immunofluorescence of treated cells show expression of phospho-c-JUN (T91/T93) in SKBR3 cells (green channel) but not on HCT116 cells. Dapi (blue channel) was used to localize the nuclei. C. Immunoblot of phospho-c-JUN (T91/T93) using SDS extracts of poorly differentiated HCT116 cells treated with 40μM of flavone B (+B), or the dissolution vehicle DMSO (-). SDS lysates from SKBR3 cells treated with 10μM tamoxifen (+) were used as a positive control. After SDS-PAGE, nitrocellulose membranes were probed with an antibody specific to this phosphorylated form. D. Detection of caspase 8 by immunoblot of SDS lysates of HCT116 cells 1.5, 3, 6, 9 and 12 hours (lanes 2–6) after treatment with flavone B or vehicle (DMSO) for the control (C, lane 1) and SDS-PAGE. The membranes were probed with an antibody capable of detecting both the procaspase (54/55 kDa) and the fragments resultant after activation (43 and 18 kDa). The membranes were reprobed for actin or tubulin as a loading control. The results shown are representative of three independent experiments.

To confirm flavone B activation of the apoptotic program via the extrinsic pathway without the involvement of the intrinsic pathway, we studied phosphorylation forms of c-JUN relevant to the engagement of the mitochondria via the cleavage of caspase 8 [25, 26]. HCT 116 and MIA PaCa cells dosed with flavone B do not show activation of c-JUN on threonines 91 and 93 as shown in Fig 6A via immunofluorescence in HCT 116 cells. Breast cancer SKBR3 cells dosed with tamoxifen, a positive control for this activation [27], were processed in the same manner as described above (Fig 6B). This result was confirmed by immunoblot as shown in Fig 6C. Examination of the activation status of caspases 8 and 10 in these cells after treatment with flavone B showed no cleavage of this protein in either the HCT 116 or the MIA PaCa cells. This is evident by the presence of the uncleaved caspases at different time points spanning from 1.5–12 hours. Representative immunoblots of time course experiments for caspases 8 and 10 in HCT 116 cells are shown in Fig 6D and 6E respectively.

## Discussion

We had previously shown the differential antineoplastic effects of flavones A and B on cancers cells of the breast (MCF7, SKBR3), colon (CaCo-2, HCT116), pancreas (Panc 28, MIA PaCa), and prostate (LnCaP, PC3) with dissimilar differentiation status. The flavones were extracted from *G*. *elegans* and *A*. *bogotensis*, plants with like medicinal properties that are thus used indistinctively according to ethnobotanical studies. However, the flavones are not exclusive to these species. Flavone A has been found in *Ainsliaea henryi* [28] and in *Helichrysum decumbens [29]* while Flavone B has been isolated from *Helichrysum graveolens* [30], as well as *Helichrysum odoratissimum* [31], and *Helichrysum compactum* [32]. Flavones A and B demonstrated a significant cytotoxic effect against highly tumorigenic cell lines, while sparing normal epithelial cells. Specifically, flavone A demonstrated high cytotoxicity against the more differentiated Panc28 and CaCo-2 cells, while flavone B showed a preference for poorly differentiated MIA PaCa, HCT 116, and SKBR3 cells [20]. Cell doubling time, and the presence of specific differentiation and polarity markers are used to determine cellular differentiation status. Among the cells we tested, Panc28 has been previously described as poorly differentiated mainly due to the absence of polarity markers present in other pancreatic cell lines such as Capan-1, despite the fact that it has a fairly long cell doubling time. However, it is the general consensus that Panc 28 cells have a higher differentiation status than MIA PACa cells [33], and our results suggest that the flavones may be sensitive to this difference. To better understand the mechanism by which the apoptotic program is initiated by flavones A and B in their target cells, we assessed the effect of each flavone on key extrinsic and intrinsic apoptotic pathway proteins, such as ERK, pS6, AKT, BAD, and c-JUN in their activated forms.

Our results suggest that Flavone A may induce apoptosis in better differentiated pancreatic and colon cancer cells, via the mitochondrial intrinsic pathway. This is evident by the decrease in phosphorylated ERK and S6 and subsequent loss of activated BAD. Phosphorylation keeps BAD in the cytoplasm, and its loss results in binding and inactivation of the survival proteins Bcl-xL or Bcl-2 after crossing the mitochondrial membrane [34] thereby activating apoptosis. A significant decrease of phosphorylated BAD at serine 112 is observed in pancreatic cancer Panc 28 and colon cancer CaCo-2 cells after treatment with flavone A. The activation of BAD at serine 112 is mediated by the MAPK pathway, specifically via the activation of Ras-Raf-ERK [35]. Thus, the inhibition of phosphorylated ERK and S6 observed after treatment with flavone A, could be upstream of the loss of activated BAD at serine 112 and the subsequent initiation of apoptosis via the disruption of mitochondrial membrane integrity and the release of cytochrome c and other apoptotic factors. These events may lead to the activation of caspase 9 and effector caspases. However, caspases are highly deregulated in cancer through various mutations or loss of expression[36]. In the case of caspase 9, these are uncommon, and it is the abrogation of post mitochondrial functional activities that favor tumor progression[37]. This may be the case in our caspase 9 results, where activated caspase is present in control and treated cells, which may suggest loss of apoptotic function. It is important to note that impaired mitochondrial integrity can trigger a default caspase 9-independent program of cell death[38]. Whether apoptosis is occurring independently of caspase 9 activity or by the creation of a favorable ratio of active vs inactive caspase, as seen in our results, we present evidence that support the activation of an intrinsic pathway. Additionally, our results also show that neither AKT nor c-JUN are involved in the proposed mechanism of action for Flavone A.

Conversely, flavone B induces apoptosis in poorly differentiated cancer cells of the pancreas and the colon via an extrinsic pathway. Our results show concomitant upregulation of the activated forms of ERK and c-JUN after treatment of these cells with Flavone B. Previous studies have demonstrated that the activation of ERK and c-JUN regulate the transcriptional activity of activator protein 1 (AP-1), and subsequently lead the cell to apoptotic death [39]. Essential to the formation of the AP-1 transcription factor in this apoptotic mechanism is the phosphorylation of c-JUN at threonines 91 and 93 [27, 40]. We have shown in this study that this activation is not present after dosing the cells with flavone B, supporting the activation of apoptosis without the involvement of AP-1. Besides contributing to the onset of apoptosis, the upregulation of phosphorylated ERK is likely to support the presence of BAD in the cytoplasm, via its sustained activation at serine 112, as shown in our results. It is also possible that the anti-apoptotic function of phosphorylated BAD involves activation at a different site, such as serine 136. However, this activation is mediated via AKT[41], a protein that remained unchanged in our analysis of the mechanism of action of Flavone B. Alternatively, caspase 8 could provide crosstalk [25] between the extrinsic and the intrinsic pathways by engaging the mitochondria, and eliciting a pro-apoptotic response via the release of cytochrome C. However, no change was observed on the status of caspase 8 in these cells after treatment with Flavone B, thus no fragments were detected (Fig 5D). Caspase 8 is recognized as the predominant initiator caspase in the extrinsic pathway. Caspase 10 has also been associated with the extrinsic pathway, but its mechanisms of activation are still not completely elucidated. Since a recent report has shown that caspase 10 was also able to engage the mitochondria[42], we tested caspase 10 in the same manner as caspase 8 and obtained similar results after treatment with flavone B. We have also found dissimilar effects on these cell lines for the expression levels of S6 which is thought to be involved in protein translation and cell proliferation. Thus, this data may indicate downregulation of protein expression in MIA PaCa cells and an increase in HCT 116 cells. Since flavone B ultimately causes inhibition of cellular viability on both cell lines, this effect may be related to specific cell type, as the cells respond to the interaction with the flavone.

All together, these results provide evidence of the signaling involved in the preferential cytotoxic activities of these isomers. Not only are the underlying mechanism and targets different, but the type of pathways by which the two flavones induce apoptosis vary as well. This may allude to the possible benefits of combining flavone A and flavone B for increased effectiveness and may hold a key to a novel treatment or treatment target in cancer therapy. We have previously reported the EC50 of flavone A and flavone B to be 51.76 and 33.18 μM against pancreatic cancers, and 12.42 and 74.82 μM against colon cancers respectively[20]. Similar in vitro experiments using currently used chemotherapy drugs for these malignancies, report concentrations ranges of 0.05–9μM for gemcitabine [43, 44] and 5–50 μM for 5-fluorouracil [45]. It is important to note that the mechanisms by which these drugs inhibit cell viability are quite different and translate into longer time points to allow detection of loss of cell viability. Both chemotherapy drugs induce DNA damage, and suppression of viability is apparent 48–72 hours after dosing. The flavones however, inhibit cell survival within 24 hours. It is possible that the mechanisms of these two types of drugs could work together to enhance effectiveness against these malignancies.

While these results suggest that the two flavones in this study effectively initiate apoptosis in these tumorigenic cells, the chemical properties of these two isomers dictate preferential interactions of these compounds with proteins within the cell; thus, more studies are warranted to completely elucidate these relationships.

## Supporting Information

S1 Fig Molecular structures of flavones A and B. A. Flavone A was identified by its physical and spectroscopic properties as 5,7 dihydroxy-3,6,8 trimethoxyflavone. B. Flavone B was identified by its physical and spectroscopic properties as 3,5-dihydroxy-6,7,8-trimethoxyflavone.
(TIF)

## References

[1] Jemal A, Bray F, Center MM, Ferlay J, Ward E, Forman D. Global cancer statistics. CA Cancer J Clin. 2011;61(2):69–90. Epub 2011/02/08. doi: caac.20107 [pii] 10.3322/caac.20107 . 21296855

[2] Newman DJ, Cragg GM. Natural products as sources of new drugs over the last 25 years. J Nat Prod. 2007;70(3):461–77. 10.1021/np068054v . 17309302

[3] Chiang LC, Ng LT, Lin IC, Kuo PL, Lin CC. Anti-proliferative effect of apigenin and its apoptotic induction in human Hep G2 cells. Cancer Lett. 2006;237(2):207–14. 10.1016/j.canlet.2005.06.002 . 16023288

[4] Dandawate PR, Vyas A, Ahmad A, Banerjee S, Deshpande J, Swamy KV, et al Inclusion complex of novel curcumin analogue CDF and beta-cyclodextrin (1:2) and its enhanced in vivo anticancer activity against pancreatic cancer. Pharm Res. 2012;29(7):1775–86. 10.1007/s11095-012-0700-1 22322899 PMC3868989

[5] Lopez-Lazaro M. Flavonoids as anticancer agents: structure-activity relationship study. Curr Med Chem Anticancer Agents. 2002;2(6):691–714. . 12678721 10.2174/1568011023353714

[6] Kandaswami C, Lee LT, Lee PP, Hwang JJ, Ke FC, Huang YT, et al The antitumor activities of flavonoids. In Vivo. 2005;19(5):895–909. . 16097445

[7] Kale A, Gawande S, Kotwal S. Cancer phytotherapeutics: role for flavonoids at the cellular level. Phytother Res. 2008;22(5):567–77. 10.1002/ptr.2283 . 18398903

[8] Wang HK. The therapeutic potential of flavonoids. Expert Opin Investig Drugs. 2000;9(9):2103–19. 10.1517/13543784.9.9.2103 . 11060796

[9] Tseng TH, Lee YJ. Evaluation of natural and synthetic compounds from East Asiatic folk medicinal plants on the mediation of cancer. Anticancer Agents Med Chem. 2006;6(4):347–65. . 16842235 10.2174/187152006777698150

[10] Choudhary MI, Hareem S, Siddiqui H, Anjum S, Ali S, Atta Ur R, et al A benzil and isoflavone from Iris tenuifolia. Phytochemistry. 2008;69(9):1880–5. 10.1016/j.phytochem.2008.03.011 . 18472117

[11] Javed H, Khan MM, Ahmad A, Vaibhav K, Ahmad ME, Khan A, et al Rutin prevents cognitive impairments by ameliorating oxidative stress and neuroinflammation in rat model of sporadic dementia of Alzheimer type. Neuroscience. 2012;210:340–52. 10.1016/j.neuroscience.2012.02.046 . 22441036

[12] Garcia-Lafuente A, Guillamon E, Villares A, Rostagno MA, Martinez JA. Flavonoids as anti-inflammatory agents: implications in cancer and cardiovascular disease. Inflamm Res. 2009;58(9):537–52. 10.1007/s00011-009-0037-3 . 19381780

[13] Cushnie TP, Lamb AJ. Antimicrobial activity of flavonoids. Int J Antimicrob Agents. 2005;26(5):343–56. . 16323269 10.1016/j.ijantimicag.2005.09.002 PMC7127073

[14] Zandi K, Teoh BT, Sam SS, Wong PF, Mustafa MR, Abubakar S. Novel antiviral activity of baicalein against dengue virus. BMC Complement Altern Med. 2012;12:214 10.1186/1472-6882-12-214 23140177 PMC3528482

[15] Visintini Jaime MF, Redko F, Muschietti LV, Campos RH, Martino VS, Cavallaro LV. In vitro antiviral activity of plant extracts from Asteraceae medicinal plants. Virol J. 2013;10:245 10.1186/1743-422X-10-245 23890410 PMC3733733

[16] Torrenegra RD, Pedrozo P, Rojas C, Carrizosa S. Plantas Colombianas del ge´nero Gnaphalium (IV) G. rufescens y G. antennarioides. Revista Latinoamericana de Quı´mica. 1987;18:116–8.

[17] Garcia-Barriga H. Flora Medicinal de Colombia Bogota, Colombia: Imprenta Nacional III; 1975.

[18] Torrenegra RD, Escarria S, Raffelsberger B, Achenbach H. 5,7-Dihydroxy-3,6,8-trimethoxyflavone from the flowers of Gnaphalium elegans. Phytochemistry. 1980;19:2795–6.

[19] Torrenegra RD, Escarria S, Tenorio E, Achenbach H. Estudio fitoquimico del Achirocline bogotensis. Rev Latinoam Quim. 1982;13:75–6.

[20] Thomas CM, Wood RC 3rd, Wyatt JE, Pendleton MH, Torrenegra RD, Rodriguez, et al Anti-neoplastic activity of two flavone isomers derived from Gnaphalium elegans and Achyrocline bogotensis. PLoS One. 2012;7(6):e39806 10.1371/journal.pone.0039806 22768128 PMC3387256

[21] Neumeister V, Agarwal S, Bordeaux J, Camp RL, Rimm DL. In situ identification of putative cancer stem cells by multiplexing ALDH1, CD44, and cytokeratin identifies breast cancer patients with poor prognosis. Am J Pathol. 2010;176(5):2131–8. 10.2353/ajpath.2010.090712 20228222 PMC2861079

[22] Lin L, Liu Y, Li H, Li PK, Fuchs J, Shibata H, et al Targeting colon cancer stem cells using a new curcumin analogue, GO-Y030. Br J Cancer. 2011;105(2):212–20. 10.1038/bjc.2011.200 21694723 PMC3142799

[23] Visus C, Wang Y, Lozano-Leon A, Ferris RL, Silver S, Szczepanski MJ, et al Targeting ALDH(bright) human carcinoma-initiating cells with ALDH1A1-specific CD8(+) T cells. Clin Cancer Res. 2011;17(19):6174–84. 10.1158/1078-0432.CCR-11-1111 21856769 PMC3186874

[24] Pfister AB, Wood RC, Salas PJ, Zea DL, Ramsauer VP. Early response to ErbB2 over-expression in polarized Caco-2 cells involves partial segregation from ErbB3 by relocalization to the apical surface and initiation of survival signaling. J Cell Biochem. 2010;111(3):643–52. 10.1002/jcb.22754 20589763 PMC3075438

[25] Li H, Zhu H, Xu CJ, Yuan J. Cleavage of BID by caspase 8 mediates the mitochondrial damage in the Fas pathway of apoptosis. Cell. 1998;94(4):491–501. . 9727492 10.1016/s0092-8674(00)81590-1

[26] Luo X, Budihardjo I, Zou H, Slaughter C, Wang X. Bid, a Bcl2 interacting protein, mediates cytochrome c release from mitochondria in response to activation of cell surface death receptors. Cell. 1998;94(4):481–90. . 9727491 10.1016/s0092-8674(00)81589-5

[27] Madeo A, Vinciguerra M, Lappano R, Galgani M, Gasperi-Campani A, Maggiolini M, et al c-Jun activation is required for 4-hydroxytamoxifen-induced cell death in breast cancer cells. Oncogene. 2010;29(7):978–91. 10.1038/onc.2009.400 . 19935718

[28] Xiong HP, Wu ZJ, Chen FT, Chen WS. 5,7-Dihydr-oxy-3,6,8-trimethoxy-flavone. Acta Crystallogr Sect E Struct Rep Online. 2009;65(Pt 12):o3276–7. 10.1107/S1600536809050715 21578970 PMC2971965

[29] Toma´s-Lorente F, I-Sn E, Toma´s-Barbera´n FA, Trowitzsch-W K, V W. Antifungal phloroglucinol derivatives and lipophilic flavonoids from Helichrysum decumbens. Phytochemistry. 1989;28:1613–5.

[30] Hansel R, B C. 3,5-Dihydroxy-6,7,8-trimethoxyflavon aus Helichrysum graveolens. Phytochemistry. 1972;11:26–32.

[31] I VP, N DK, J C, Munyjabo V, S N. Isolation of flavonoids and a chalcone from Helichrysum odoratissimum and synthesis of helichrysetin. J Nat Prod. 1989;52:629–33. 2778452 10.1021/np50063a025

[32] Suzgec S, Mericli AH, Houghton PJ, Cubukcu B. Flavonoids of Helichrysum compactum and their antioxidant and antibacterial activity. Fitoterapia. 2005;76(2):269–72. 10.1016/j.fitote.2004.12.006 . 15752647

[33] Sipos B, Moser S, Kalthoff H, Torok V, Lohr M, Kloppel G. A comprehensive characterization of pancreatic ductal carcinoma cell lines: towards the establishment of an in vitro research platform. Virchows Arch. 2003;442(5):444–52. 10.1007/s00428-003-0784-4 . 12692724

[34] Yang E, Zha J, Jockel J, Boise LH, Thompson CB, Korsmeyer SJ. Bad, a heterodimeric partner for Bcl-XL and Bcl-2, displaces Bax and promotes cell death. Cell. 1995;80(2):285–91. . 7834748 10.1016/0092-8674(95)90411-5

[35] Fang X, Yu S, Eder A, Mao M, Bast RC Jr., Boyd D, et al Regulation of BAD phosphorylation at serine 112 by the Ras-mitogen-activated protein kinase pathway. Oncogene. 1999;18(48):6635–40. 10.1038/sj.onc.1203076 . 10597268

[36] Philchenkov A, Zavelevich M, Kroczak TJ, Los M. Caspases and cancer: mechanisms of inactivation and new treatment modalities. Exp Oncol. 2004;26(2):82–97. . 15273659

[37] Olsson M, Zhivotovsky B. Caspases and cancer. Cell Death Differ. 2011;18(9):1441–9. 10.1038/cdd.2011.30 21455218 PMC3178435

[38] van Delft MF, Smith DP, Lahoud MH, Huang DC, Adams JM. Apoptosis and non-inflammatory phagocytosis can be induced by mitochondrial damage without caspases. Cell Death Differ. 2010;17(5):821–32. 10.1038/cdd.2009.166 19911005 PMC3005563

[39] Xu C, Shen G, Yuan X, Kim JH, Gopalkrishnan A, Keum YS, et al ERK and JNK signaling pathways are involved in the regulation of activator protein 1 and cell death elicited by three isothiocyanates in human prostate cancer PC-3 cells. Carcinogenesis. 2006;27(3):437–45. 10.1093/carcin/bgi251 . 16272172

[40] Vinciguerra M, Esposito I, Salzano S, Madeo A, Nagel G, Maggiolini M, et al Negative charged threonine 95 of c-Jun is essential for c-Jun N-terminal kinase-dependent phosphorylation of threonine 91/93 and stress-induced c-Jun biological activity. Int J Biochem Cell Biol. 2008;40(2):307–16. 10.1016/j.biocel.2007.08.001 . 17920329

[41] Datta SR, Dudek H, Tao X, Masters S, Fu H, Gotoh Y, et al Akt phosphorylation of BAD couples survival signals to the cell-intrinsic death machinery. Cell. 1997;91(2):231–41. . 9346240 10.1016/s0092-8674(00)80405-5

[42] Wachmann K, Pop C, van Raam BJ, Drag M, Mace PD, Snipas SJ, et al Activation and specificity of human caspase-10. Biochemistry. 2010;49(38):8307–15. 10.1021/bi100968m 20795673 PMC2943529

[43] Yeo D, Huynh N, Beutler JA, Christophi C, Shulkes A, Baldwin GS, et al Glaucarubinone and gemcitabine synergistically reduce pancreatic cancer growth via down-regulation of P21-activated kinases. Cancer Lett. 2014;346(2):264–72. 10.1016/j.canlet.2014.01.001 . 24491405 PMC7681251

[44] Rathos MJ, Joshi K, Khanwalkar H, Manohar SM, Joshi KS. Molecular evidence for increased antitumor activity of gemcitabine in combination with a cyclin-dependent kinase inhibitor, P276-00 in pancreatic cancers. J Transl Med. 2012;10:161 10.1186/1479-5876-10-161 22873289 PMC3478973

[45] Gonzalez-Vallinas M, Molina S, Vicente G, de la Cueva A, Vargas T, Santoyo S, et al Antitumor effect of 5-fluorouracil is enhanced by rosemary extract in both drug sensitive and resistant colon cancer cells. Pharmacol Res. 2013;72:61–8. 10.1016/j.phrs.2013.03.010 . 23557932

