# Supplementary figures and images for: Mechanism of Action of Two Flavone Isomers Targeting Cancer Cells with Varying Cell Differentiation Status
Source: PLoS One. 2015 Nov 25;10(11):e0142928. doi: 10.1371/journal.pone.0142928 (PMC4659548; doi:10.1371/journal.pone.0142928)

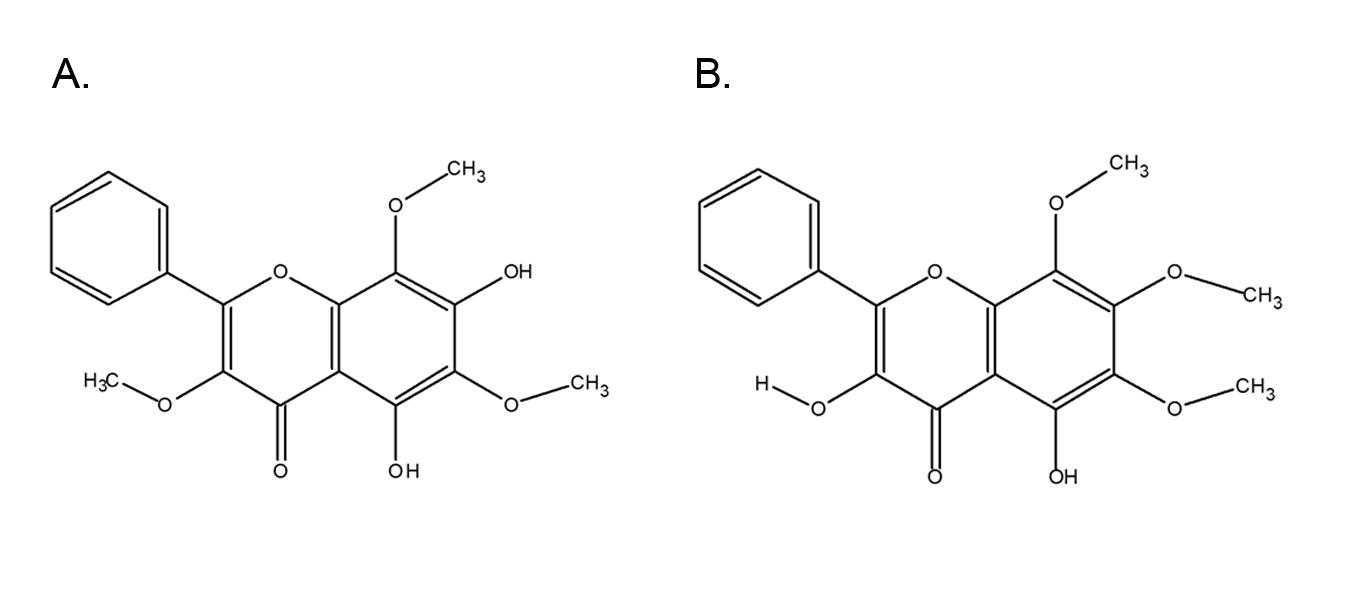

Supplement: S1 Fig — A. Flavone A was identified by its physical and spectroscopic properties as 5,7 dihydroxy-3,6,8 trimethoxyflavone. B. Flavone B was identified by its physical and spectroscopic properties as 3,5-dihydroxy-6,7,8-trimethoxyflavone. (TIF) [file pone.0142928.s001.tif]
